# A Novel Homozygous Nucleotide Deletion in the JAK2 Gene in a Pediatric Patient with B-cell Precursor Acute Lymphoblastic Leukemia

**DOI:** 10.5505/tjh.2012.44712

**Published:** 2012-06-15

**Authors:** Dilara Fatma Akın, Emel Akkaya, A. Emin Kürekçi, Çiğdem Arslan, Üstün Ezer, Nejat Akar

**Affiliations:** 1 LOSEV Foundation for Children with Leukemia, Ankara, Turkey; 2 Ankara University, Department of Pediatric Genetics, Ankara, Turkey

## TO THE EDITOR

Janus kinase 2 (JAK2) is a protein tyrosine kinase that transduces cellular signals through the Janus kinase signal transducer and an activator of transcription pathways (JAK-STAT), which mediates cell growth, differentiation, apoptosis, transformation, and other fundamental cell functions, and is active in both normal hematopoiesis and hematological malignancies. Tyrosine kinase protein mutations are important in the development of malignant processes. JAK2 mutations have been identified in myeloproliferative disorders, of which exon 14 V617F mutation is the most common. Other novel mutations were identified in exon 12, which is located at the proximal region of the gene. These reported mutations impair the organization of JAK2 kinase activity [1,2,3,4]. Herein, we report a novel homozygous “G” deletion at exon 12 of the JAK2 gene in a pediatric patient with acute lymphoblastic leukemia (ALL).

## CASE

A 20-month-old male patient with persistent high fever, cough, and diarrhea was admitted to the hospital. Physical examination was normal, except for hepatomegaly. Complete blood count showed pancytopenia with 40% lymphoblasts in his peripheral blood smear. Bone marrow smear examination showed L1-type diffuse lymphoblastic infiltration. Precursor B-cell ALL was the final diagnosis based on flow cytometric analysis; there were no additional cytogenetic aberrations. Chromosomal analysis showed that the patient was 46 XY. The ALL Berlin-Frankfurt- Munster (ALL-BFM 95) standard-risk protocol was initiated. During the treatment there were no problems and the first remission was achieved on the 33rd d of chemotherapy. At the time this manuscript was written, the patient was still in first remission and continuing maintenance therapy. 

**Figure 1:**
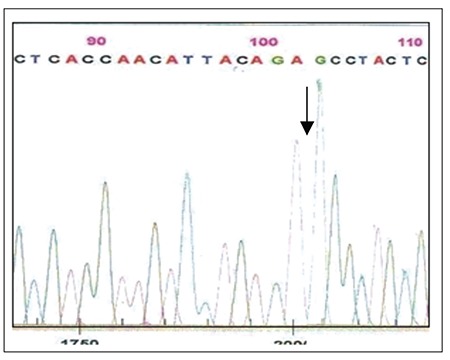
Sequencing analysis of exon 12 of the JAK2 gene inthe presented case at admission shows deletion of 1584 del G.

**Figure 2:**
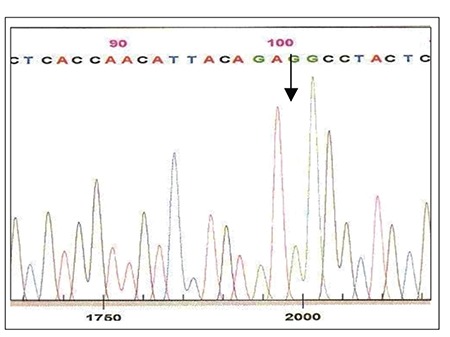
Sequencing analysis of exon 12 of the JAK2 gene in the presented case while in remission.

Blood samples were obtained from the patient and his mother at admission and remission, and the phenolchloroform method was used to extract DNA. Written informed consent was obtained from the parents of patient. Using primers f5’CTCCTCTTTGGAGCAATTCA3’ and r5’TATCGCAACTCCCAAGTTCTC3’ we amplified and sequenced exon 12 of the JAK2 gene (Beckman Coulter, USA). All sequencing reactions were performed twice, using 2 different PCR products. Sequencing of the exon at admission showed that a homozygous “G” deletion at nt 2078 (in genomic seq./nt 1584 in c-DNA seq.) resulted in substitution of arginine (AGG) with serine (AGC) in codon 528 (Figures 1 and 2) (ref Seq; ENSG00000096968). In all, we scanned 14 patients with ALL.

The observed substitution was a novel change. We did not observe “G” deletion in the patient during remission or in his mother. A Western blot showing a truncated protein would have significantly strengthened our report; however, serum sample at admission was unavailable. The observed mutation affecting the amino acid sequence and disturbing the regulation of JAK2 kinase activity may be a factor expected to cause extreme sensitivity to erythropoietin. Although it was reported that JAK2 V617F mutation is absent in childhood ALL, rare mutations have been reported, especially in Down syndrome patients with acute leukemia [4,5,6,7,8,9]. A 5-base deletion in the region was first reported in a Down syndrome patient associated with B-cell precursor ALL [7]. Moreover it was recently reported that CRLF2 gene rearrangements were strongly associated with JAK2 mutations [10].

The diagnosis of B-cell precursor ALL in the presented case warrants comprehensive mutational screening of the entire JAK2 gene coding exon in patients with this type of ALL. 

**Conflict of Interest Statement **

The authors of this paper have no conflicts of interest, including specific financial interests, relationships, and/ or affiliations relevant to the subject matter or materials included.
